# Help for Armenia

**Published:** 1922-07

**Authors:** Emily J. Robinson

**Affiliations:** Hon. Secretary, Armenian Red Cross and Refugee Fund. 35a, Elsham Road, Kensington, W. 14


*To the Editor of* THE HOSPITAL AND HEALTH REVIEW.

Yours faithfully,

F. SQUIRES.

### Help for Armenia.

*To the Editor of* THE HOSPITAL AND HEALTH REVIEW.

SIR,—Having been asked by the Red Cross Society of Armenia at Erivan to act as their representative in Great Britain and the Colonies to collect funds and supply the drugs and hospital requisites so badly wanted, I beg to send you an extract from a report which has just reached me from the Red Cross Committee at Erivan :—

" From time immemorial malaria has been a widely spread disease amongst the population of Armenia, and now it is the most menacing epidemic threatening them ; 15 per cent. of the population are subject to that disease." The committee recommend that in malaria centres sanitary stations should be established, that if possible 1,000 kilos of quinine should be distributed free to the people, that swamps should be drained, a pure water supply ensured, &c. The malaria takes a pernicious form. Strong men rapidly become unconscious and succumb unless an injection of quinine can be given.

The people of our smallest ally are working bravely to help themselves, but every necessary of life is lacking in Armenia. Over 700,000 persons are existing on rations provided by relief funds. I shall be very thankful to receive donations in money for the hospitals or for the purchase of quinine, or offers of gifts of quinine or salvarsan or hospital requisites, which can be sent to Armenia promptly.

Yours faithfully,
EMILY J. ROBINSON,
Hon. Secretary,
Armenian Red Cross and Refugee Fund.
35A, Elsham Road,
Kensington, W. 14.

